# ‘Spin’ in published biomedical literature: A methodological systematic review

**DOI:** 10.1371/journal.pbio.2002173

**Published:** 2017-09-11

**Authors:** Kellia Chiu, Quinn Grundy, Lisa Bero

**Affiliations:** Charles Perkins Centre, Faculty of Pharmacy, The University of Sydney, Sydney, New South Wales, Australia; Université Paris Descartes, France

## Abstract

In the scientific literature, spin refers to reporting practices that distort the interpretation of results and mislead readers so that results are viewed in a more favourable light. The presence of spin in biomedical research can negatively impact the development of further studies, clinical practice, and health policies. This systematic review aims to explore the nature and prevalence of spin in the biomedical literature. We searched MEDLINE, PreMEDLINE, Embase, Scopus, and hand searched reference lists for all reports that included the measurement of spin in the biomedical literature for at least 1 outcome. Two independent coders extracted data on the characteristics of reports and their included studies and all spin-related outcomes. Results were grouped inductively into themes by spin-related outcome and are presented as a narrative synthesis. We used meta-analyses to analyse the association of spin with industry sponsorship of research. We included 35 reports, which investigated spin in clinical trials, observational studies, diagnostic accuracy studies, systematic reviews, and meta-analyses. The nature of spin varied according to study design. The highest (but also greatest) variability in the prevalence of spin was present in trials. Some of the common practices used to spin results included detracting from statistically nonsignificant results and inappropriately using causal language. Source of funding was hypothesised by a few authors to be a factor associated with spin; however, results were inconclusive, possibly due to the heterogeneity of the included papers. Further research is needed to assess the impact of spin on readers’ decision-making. Editors and peer reviewers should be familiar with the prevalence and manifestations of spin in their area of research in order to ensure accurate interpretation and dissemination of research.

## Author summary

In the scientific literature, spin refers to reporting practices that distort the interpretation of results and mislead readers so that results are viewed in a more favourable light. The presence of spin in biomedical research can negatively impact the development of further studies, clinical practice, and health policies. We conducted a systematic review to explore the nature and prevalence of spin in the biomedical literature. We included 35 reports, which investigated spin in clinical trials, observational studies, diagnostic accuracy studies, systematic reviews, and meta-analyses. The nature of spin varied according to study design. The highest (but also greatest) variability in the prevalence of spin was present in trials. Some of the common practices used to spin results included detracting from statistically nonsignificant results and inappropriately using causal language. Source of funding was hypothesised by a few authors to be a factor associated with spin; however, results were inconclusive, possibly due to the heterogeneity of the included papers. Further research is needed to assess the impact of spin on readers’ decision-making. Editors and peer reviewers should be familiar with the prevalence and manifestations of spin in their area of research in order to ensure accurate interpretation and dissemination of research.

## Introduction

Spin, commonly associated with propaganda, public relations, and the media, is broadly understood as a biased presentation, intended to ensure that audiences view matters favourably. Spin also occurs in published biomedical research, sometimes known as ‘science hype’, where scientific findings are inappropriately overstated [1]. In the scientific literature, spin refers to specific reporting practices that distort the interpretation of results and mislead readers so that results are viewed in a more favourable light [2].

Accurate reporting and interpretation of research results is essential for knowledge translation and has implications for the development of further studies, policies, and clinical practice. Examples of spin include misinterpreting statistically nonsignificant results as ‘showing an effect’ or the selective interpretation of results to emphasise significant secondary outcomes and minimizing nonsignificant primary outcomes [2]. These tactics could lead to subsequent research on clinical interventions for which there is a lack of supporting evidence. This, in turn, could lead to skewed systematic reviews and misinformed clinical practice guidelines or health policies. In addition, ‘promising’ scientific discoveries that are based upon conclusions with spin rather than data could stimulate financial investments in medical interventions that are later found to be ineffective or even harmful [1].

Spin is an enduring topic in research [3]; however, there has been recent interest in spin in the reporting and interpretation of results in published biomedical research. Boutron et al. [2] defined spin as ‘specific reporting strategies, whatever their motive, to highlight that the experimental treatment is beneficial, despite a statistically non-significant difference of the primary outcome, or to distract the reader from statistically non-significant results.’ This definition has served as a basis for other researchers investigating spin in published studies in particular clinical fields [4–8]. However, to date, there has been no systematic review or meta-analysis of the nature or prevalence of spin in biomedical literature in general or across study designs. Thus, neither the extent of spin nor its implications are well understood.

The objectives of this methodological systematic review were to examine the nature, prevalence and implications of spin in published biomedical literature across disciplines and clinical areas. The research questions included: How has spin been studied in the biomedical literature? How does spin manifest and what is its prevalence? What factors are associated with the presence of spin? Although we defined the population of interest (empirical biomedical publications) and exposure (spin) a priori, we included all spin-related outcomes reported in the identified sample of reports. As a number of studies hypothesised that funding source was a factor associated with spin, we tested this hypothesis in our review.

## Results

### Characteristics of included reports

**Fig 1:**
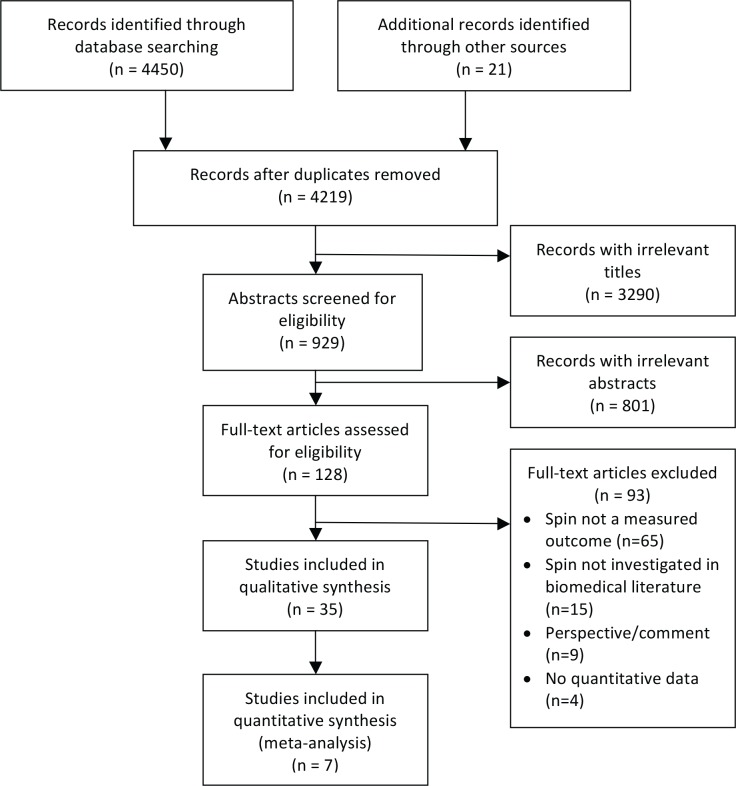
PRISMA flowchart of included articles.

A total of 4,471 reports were identified, with 4,450 acquired through searching the electronic databases and 21 through hand-searching the reference lists of included reports. A flowchart of the screening process is summarised in Fig 1, and Table 1 shows the characteristics of the included reports. Of the 35 included reports, 22 (63%) were published in the last 5 years (since 2012), and 34 (97%) were published in the last 10 years (since 2007). The majority of reports (31/35, 89%) were reviews of published literature designed to assess the occurrence of spin in published biomedical literature; other designs included a survey (1/35, 3%), a randomised controlled trial (RCT) (1/35, 3%) designed to assess the effects of spin, and examination of data sources such as regulatory or company documents (2/35, 6%). The majority of reports (18/35, 51%) received funding from public or not-for-profit sources; 10 reports (10/35, 29%) did not disclose their funding source. Sixteen reports (16/35, 46%) declared that authors had no conflicts of interest; 6 of the reports (6/35, 17%) did not make a disclosure statement.

**Table 1:** Characteristics of included reports (*n* = 35).

Study (author, year [reference])	Study design^1^	*n*	Field of research of included studies	Study design of included studies	Time frame of included studies	Study funding source	Author conflict of interest
Alasbali, 2009 [9]	Review	39	Topical prostaglandin analogues	Meta-analysis; RCT; nonrandomised controlled trial	2001 to 2007	None	Yes
Altwairgi, 2012 [10]	Review	114	Systemic therapy in lung cancer	RCT	2004 to 2009	Not disclosed	No
Arunachalam, 2016 [11]	Review	110	Surgical trials	RCT	2013 to 2015	None	No
Boutron, 2010 [2]	Cross-sectional	72	Not restricted	RCT	Dec 2006	Government	No
Boutron, 2014 [12]	RCT	30	Oncology	RCT	2005 to 2009	Government; not-for-profit organisation	Yes
Brody, 2013 [13]	Cross-sectional	15	Surgical trials	Trials	2008	Government	No
Brown, 2013 [14]	Review	88	Obesity	Observational	Not stated	Government	Yes
Cofield, 2010 [15]	Cross-sectional	161	Obesity and nutrition	Observational	2006	Government	Yes
Cordoba, 2010 [16]	Cross-sectional	40	Not restricted	Parallel group RCT	2008	None	No
Djulbegovic, 2011 [17]	Systematic review	374	Oncology	RCT	1955 to 2006	Government	No
Fernandez Y Garcia, 2010 [18]	Review (longitudinal analysis)	87	Not restricted	RCT	1994, 1999, and 2004	Industry; government	Yes
Gewandter, 2015 [19]	Review	76	Analgesics	RCT	2006 to 2013	Industry; not-for-profit organisation	Yes
Hernandez, 2013 [20]	Systematic review	16	Antiretroviral therapy	Noninferiority RCT	2000 to 2012	Not-for-profit organisation	Yes
Jefferson, 2009 [21]	Systematic review	274	Influenza vaccines	RCT; controlled clinical trial; cohort; case-control	Not stated (up to 2006)	Government	Yes
Latronico, 2013 [22]	Review	111	Intensive care	RCT	2001 to 2010	Not disclosed	Not disclosed
Lazarus, 2015 [4]	Review	128	Not restricted	Nonrandomised trials	2011 to 2013	Not-for-profit organisation	No
Le Fourn, 2013 [23]	Review	12	Pharmacological treatment of autoimmune or idiopathic chronic urticaria	RCT	1996 to 2011	Not-for-profit organisation	No
Li, 2009 [24]	Review	73	Quality improvement interventions	Experimental or observational	2002 to 2003	Not disclosed	Not disclosed
Lieb, 2016 [25]	Review	95	Psychological therapies for anxiety, depressive, or personality disorders	Systematic review	2010 to 2013	University	Yes
Lockyer, 2013 [5]	Review	71	Interventions for foot, leg, or pressure ulcers	RCT	2004 to 2009	Government	No
Lumbreras, 2009 [26]	Cross-sectional	15	Molecular diagnostic tests	Diagnostic accuracy study	2006	Government	No
Mathieu, 2012 [27]	Review	105	Rheumatology	RCT	2006 to 2008	Not disclosed	No
Ochodo, 2013 [6]	Cross-sectional	126	Not restricted	Diagnostic accuracy study	2010	Not disclosed	No
Patel, 2013 [28]	Review	58	Laparoscopic lower GI surgery	RCT	1992 to 2012	Not disclosed	None
Patel, 2015 [29]	Review	38	Robotic colorectal surgery	RCT, observational	1992 to 2014	Not disclosed	Yes
Pocock, 1987 [30]	Review	130	Not restricted	Controlled clinical trial	1985 to 1986	Not disclosed	Not disclosed
Prasad, 2013 [7]	Review	167	Not restricted	Observational study	2010	Not disclosed	No
Roest, 2015 [31]	Review (with meta-analysis)	16	Second generation antidepressants in treatment of anxiety disorders	Phase 2 and 3 RCT	1995 to 2009	Not-for-profit organisation	Yes
Tricco, 2009 [32]	Cross-sectional	296	Not restricted	Systematic review	2004	Not-for-profit organisation	Yes
Vedula, 2012 [33]	Case study	12	Off-label uses of gabapentin	RCT	1987 to 2008	University	Yes
Vera-Badillo, 2013 [34]	Review	92	Breast cancer	RCT	1995 to 2011	Not disclosed	No
Wilson, 2011 [35]	Review	10	Implantable cardioverter defibrillators	Primary prevention trial	1996 to 2009	Not disclosed	Not disclosed
Yank, 2007 [36]	Retrospective cohort	124	Antihypertensive drugs	Meta-analysis	1983 to 2004	Not-for-profit organisation; university	No
Yavchitz, 2016 [8]	Instrument development; survey	122	N/A	Systematic reviews and meta-analyses	N/A	Not-for-profit organisation; university	No
You, 2012 [37]	Review	336	Oncology (solid tumours)	RCT	2005 to 2009	Government; not-for-profit organisation	No

^1^ A systematic review was defined as having a structured, replicable, and exhaustive search strategy (no limits on year or source); a review was defined as having an ill-defined or significantly limited search strategy; a cross-sectional study was defined as sampling from a time period of 1 year or less. RCT, randomised controlled trial

The majority of the reports (23/35, 66%) investigated spin in trials. The fields of research of the included studies varied, and reports were largely focused on biomedical interventions. Eight papers (8/35, 23%) did not restrict the inclusion of studies to a clinical discipline, 5 (5/35, 14%) examined studies in oncology, and 4 (4/35, 11%) examined studies in surgery. All of the included studies were conducted with human participants.

### Methods for assessing spin

#### Defining spin

The majority of reports (30/35, 86%) defined spin a priori and then sought to assess its frequency, severity, or characteristics. There was considerable variation in how researchers defined spin. We inductively classified the ways that spin was defined into 1 of 4 categories (Table 2): (1) reporting practices that distort the interpretation of results and create misleading conclusions, suggesting a more favourable result; (2) discordance between results and their interpretation, with the interpretation being more favourable than the results; (3) attribution of causality when study design does not allow for it; and (4) overinterpretation or inappropriate extrapolation of results. Spin was defined as the inappropriate use of causal language exclusively in the context of observational or nonrandomised studies.

**Table 2:** Definitions of spin provided by the included reports (*n* = 35).

Definition	*n* = 35	Example
Reporting practices that distort the interpretation of results and create misleading conclusions, suggesting a more favourable result	20 (57%)	‘Specific reporting strategies, whatever their motive, to highlight that the experimental treatment is beneficial, despite a statistically nonsignificant difference for the primary outcome, or to distract the reader from statistically nonsignificant results’. [2] ‘We considered spin to exist when we observed an explicit description of spinning study findings in the internal company documents or a description in the main publication that appeared to re-frame the study results in order to explain away unfavorable findings or to emphasize favorable findings’. [33]
Discordance between results and their interpretation, with the interpretation more favourable than the results	9 (26%)	‘…whether data presented in the study supported the author's conclusions…’ [21]
Attribution of causality when study design does not support it	3 (9%)	‘Inappropriate use of causal language in the abstracts and titles of almost one third of human observational obesity or nutrition related study reports…’ [15]
Overinterpretation or inappropriate extrapolation of results	3 (9%)	‘We defined overinterpretation as reporting of diagnostic accuracy studies that makes tests look more favorable than the results justify’. [6]

#### Outcomes measured

Investigators of included reports assessed several different outcomes related to spin. These included the prevalence of spin (31/35, 89%), the level or severity of spin (8/35, 23%), practices used to spin results (19/35, 54%), factors associated with spin (19/35, 54%), and the impact of spin on a reader’s interpretation (3/35, 9%).

#### Instruments for assessing spin

Of the reports which assessed spin in published articles (*n* = 34; 1 included report was an RCT), 32 used a prespecified, standardized data collection instrument (94%). Nine (9/34, 26%) used or adapted the instrument developed by Boutron et al. [2], which was originally developed for the assessment of spin in RCTs with nonsignificant primary outcomes, though it was applied to intervention studies more broadly. Reports assessing the level/severity of spin exclusively used the Boutron instrument [2], which was implemented in the context of RCTs with nonsignificant primary outcomes. Twenty-three reports (23/34, 68%) used an author-generated data collection instrument, though only 11 (11/34, 32%) were subject to pilot or reliability testing. One report relied on a previously published rating scale by Ridker and Torres [38], designed to assess the significance and magnitude of the intervention effect, as a means to rate discordance.

Only 4 reports (4/34, 12%) used inductive methods to assess the nature of spin, including the seminal report by Boutron et al. [2] upon which 8 other reports relied. Two others also developed instruments specifically for the assessment of spin in nonrandomised studies [4] and systematic reviews [8], though neither has yet been replicated to our knowledge. This meant that reports generally assessed spin practices that were prespecified; few conducted exploratory assessments of the nature of spin.

#### Assessing spin

Consistent with review methods, the majority of the reports (27/34, 79%; 1 report was an RCT and this did not apply) used multiple independent data extractors to assess spin, which was acknowledged to be subjective. Reports included additional methods to reduce interpretation bias, including resolving any discrepancies through discussion until consensus was reached (22/34, 65%), review of discrepancies by a third investigator (10/34, 29%), or, less commonly, blinding data extractors to the author, funding source, or journal (2/34, 6%).

Half of the reports (17/34, 50%) that assessed spin in published literature assessed spin in both the abstract and main text, 4 of which specifically compared the main text results to the abstract and/or main text conclusions as a measure of discordance. Suggesting that the consequences of spin in the abstract were more severe given that many clinicians rely on abstracts alone, 7 reports (7/34, 21%) assessed spin in the abstract only. Nine reports (9/34, 26%) assessed spin only in the main text of the article. Three reports (3/34, 9%) additionally assessed spin in the articles’ titles.

### Prevalence of spin

Thirty-one reports (31/35, 89%) measured the prevalence of spin. Table 3 shows the prevalence of spin (median and range) in the different types of studies assessed in the reports. The highest prevalence of spin was measured in the main texts of a sample of 10 implantable cardioverter defibrillator trials, which all (100%) used at least 1 rhetorical practice resulting in spin [35]. The lowest was measured in the abstracts of a sample of RCTs of systemic therapy in lung cancer, where 9.7% presented discordant conclusions from study results [10]. In general, trials showed the greatest variability in the prevalence of spin. Though small sample sizes prevented statistical comparison between groups, trials with nonsignificant primary outcomes and with higher risk of bias (i.e., nonrandomized) appeared to have a higher prevalence of spin.

**Table 3:** Prevalence of spin in studies assessed in the included reports (*n* = 31)*.

Type of study	Subtype of study	Location in study	
		Abstract Median% (Min-Max%) (*n* measures)*	Main text Median% (Min-Max%) (*n* measures) *
Trials (overall)		56.8 (9.7–83.6) (*n* = 13)	56.5 (18.8–100) (*n* = 16)
	RCTs (superiority)	16.3 (9.6–22.9) (*n* = 2)	34.5 (18.8–83.3) (*n* = 7)
	RCTs (superiority) with non-significant primary outcome	60.5 (40.0–68.1) (*n* = 5)	60.3 (35.5–71.4) (*n* = 5)
	RCTs (superiority) with composite outcome	55.0 (50.0–82.5) (*n* = 3)	No data
	RCTs (non-inferiority)	62.5 (62.5–62.5) (*n* = 1)	68.8 (68.8–68.8) (*n* = 1)
	Controlled trials (randomized and not)	75.4 (75.4–75.4) (*n* = 1)	60.8 (21.6–100.0) (*n* = 2)
	Controlled trials (randomized and not) with non-significant primary outcome	81.6 (81.6–81.6) (*n* = 1)	58.2 (40.0–76.3) (*n* = 2)
	Non-randomized trials	64.8 (46.1–83.6) (*n* = 2)	83.9 (82.1–85.6) (*n* = 2)
Observational studies		30.7 (23.9–38.6) (*n* = 3)	85.6 (85.6–85.6) (*n* = 1)
Diagnostic accuracy studies		No data	43.7 (31.0–56.5) (*n* = 2)
Systematic reviews/meta-analyses		No data	26.3 (24.2–28.4) (*n* = 2)

* Not all reports measured spin in the both the abstract and the main text; some reports contained multiple measures of spin prevalence due to multiple definitions of spin.

### Level of spin

Nine reports (9/35, 26%) examined the level or severity of spin; 8 did so in the conclusions of trials with nonsignificant or inconclusive results. These 8 reports used a measure developed by Boutron et al. [2], which defined a ‘high’ level of spin in study conclusions as: no uncertainty in the framing of conclusions, no recommendations for further trials, no acknowledgment of the statistically nonsignificant primary outcomes, and/or making recommendations to use the intervention in clinical practice. On average, the abstracts of 30% (141/474) and main text of 22% (75/346) of trials with nonsignificant results had ‘high’ levels of spin in their conclusions.

One study sought to assess the perceived severity of spin in the context of systematic reviews. Yavchitz et al. [8] invited members of the Cochrane Collaboration to rank a sample of statements from systematic reviews and meta-analyses that included spin according to their severity using a Q-sort survey. The types of spin perceived to be most severe in the context of systematic reviews were: concluding recommendations for clinical practice when not supported by the results; titles claiming the treatment is beneficial when not supported by the results; and selective reporting of or overemphasis on analysis favouring the beneficial effect of the intervention [8].

### Practices used to spin results

Nineteen reports (19/35, 54%) investigated the practices that researchers used to spin results. We inductively grouped spin practices identified across study designs in order to demonstrate the range and diversity of spin practices but also to draw generalisations about the nature of spin across study designs and clinical areas. Spin practices measured in the included studies were thematically grouped into the following 4 categories: (1) inappropriate claims; (2) inappropriate extrapolations or recommendations for clinical practice; (3) selective reporting; and (4) more robust or favourable data presentation.

#### Inappropriate interpretation given study design

Spin manifested as claims that were inappropriate or unwarranted given the study design. For example, several reports examining spin in the context of trials with nonsignificant results found that the most common spin practice was to interpret the nonsignificant results as meaning the 2 treatments were equally good when the trial was designed to show the superiority of 1 arm [11, 13, 22, 23, 28, 29, 37]. The use of causal language was identified as a specific and the most prevalent spin practice in nonrandomised or observational studies, as study designs do not permit this type of inference [4]. For example, in a sample of 128 abstracts of nonrandomised studies evaluating an intervention, the most prevalent spin practice (53% of studies) was the use of causal language, including the use of statements that suggested the outcome was a result of the intervention (e.g., ‘X increases Y’ or ‘X facilitates the rapid recovery of Y’) or tone inferring a strong result (e.g., ‘this study shows that’ or ‘the results demonstrate’) [4].

#### Inappropriate extrapolations or recommendations for clinical practice

In studies that investigated the use of particular clinical tests or treatment options, spin may present as an inappropriate extrapolation or recommendations for clinical practice when not supported by study results. Additionally, this can include expressing confidence in the test or treatment without suggesting the need for further confirmatory studies. For example, in a sample of observational studies, 56% endorsed a recommendation for clinical practice, of which 86% failed to state that an RCT should be first performed [7].

#### Selective reporting

Researchers can spin their results through selectively and strategically reporting outcomes in various places in the report. This differs from outcome reporting bias, where all of the outcomes identified in a study protocol are not reported in the study report [39]. Selective reporting resulting in spin can include the omission of nonsignificant endpoints in the conclusion or abstract that were presented in the methods and results sections or discussing only significant secondary results to distract the reader from nonsignificant or unfavourable ones [2]. For example, in a sample of wound care trials with no clear primary outcome identified in the methods section, ‘cherry picking’ of statistically significant results was commonplace, particularly between the main text and corresponding abstract: while 74% (32/43) of reports included at least 1 statistically nonsignificant outcome in the main text, only 28% (12/43) of abstracts contained at least 1 statistically nonsignificant result [5]. Similarly, in a sample of inconclusive noninferiority trials of antiretroviral therapies, authors focused on statistically significant secondary outcomes, subgroup analyses, or modified population analyses [20]. Selective reporting could also encompass the selective citation of results from external research to support the authors’ interpretation of their data [14].

#### More robust or favourable data presentation

Researchers used a variety of general spin practices to present study results as being more favourable than data warranted. In a study that examined internal pharmaceutical company documents for evidence of spin, investigators found company emails that contained explicit descriptions of attempts to spin study findings in this manner: 1 email with the subject line 'spinning Serpell' (Serpell was the lead study investigator) stated, ‘If Pfizer wants to use, present, and publish this comparative data analysis in which 2 of 5 studies compared make the overall picture look bad, how to (sic) we make it sound better than it looks on the graphs’ [33].

This category of spin included writing an overly optimistic abstract; employing an extensive rationale to explain away nonsignificance (for example, describing nonsignificant results as ‘trends’); misleadingly describing the study design (to present it as more robust); and underreporting or ruling out adverse events. For example, in a sample of diagnostic accuracy studies, one study concluded, ‘Detection of antigen in BAL using the MVista antigen appears to be a useful method. Additional studies are needed in patients with pulmonary histoplasmosis’, whereas the abstract concluded, ‘Detection of antigen in BAL fluid complements antigen detection in serum and urine as an objective test for histoplasmosis’ [6]. A variety of rhetorical practices were used in the reporting of trials of implantable cardioverter defibrillators, including failure to discuss complications (9/10, 90%), compare the risks and benefits (10/10, 100%), or mention that benefits are likely to be less in clinical practice than in the clinical trial (10/10, 100%) [35].

### Factors associated with spin

Authors of 19 reports (19/35, 54%) assessed whether particular factors were associated with the presence of spin, including (1) conflicts of interest and study funding; (2) author characteristics; (3) journal characteristics; and (4) study design and/or quality. However, the studies were largely too heterogeneous and sample sizes too small in most instances to draw conclusions.

None of the included studies consistently found any factors to be significantly associated with spin. The only factor that was significantly and positively associated with spin across several studies was having a nonsignificant primary endpoint, though we could not conduct a quantitative meta-analysis of these data due to the heterogeneity of included studies [15, 27, 34]. This finding supports researchers’ focus on assessing spin in studies with nonsignificant results described above.

#### Conflicts of interest and funding source

**Fig 2:**
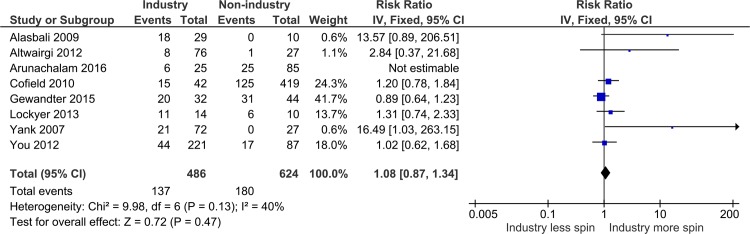
Forest plot of meta-analysis of the association between funding source and presence of spin.

Nine reports (26%) investigated the association between funding source and the presence of spin. We were able to include 7 of these (including 1,110 studies) in a meta-analysis examining the association between funding source and the presence of spin and found that industry studies were no more likely to have spin than non-industry sponsored studies (risk ratio [RR]: 1.08; 95% confidence interval [CI]: 0.87, 1.34; *I*^*2*^ = 40%) (Fig 2).

### Effects of spin on readers’ interpretation

Two reports (2/35, 6%) sought to examine the effect of spin on readers’ interpretation, though only 1 retrospectively assessed the effect on actual decision-making.

Boutron et al. [12] conducted an RCT with clinical oncology researchers to assess the effect of spin in trial abstracts on interpretation. When abstracts contained spin, readers judged the experimental treatment as more beneficial (mean difference, 0.71; 95% CI, 0.07 to 1.35; *P* = 0.030) and the trial as less rigorous (mean difference, −0.59; 95% CI, −1.13 to 0.05; *P* = 0.034) yet still were more interested in reading the full text (mean difference, 0.77; 95% CI, 0.08 to 1.47; *P* = 0.029).

Only 1 study noted an effect of spin on decision-making. Roest et al. [31] compared published articles on second-generation antidepressants for anxiety with their corresponding United States Food and Drug Administration (FDA) reviews and found that, for the not-positive trials containing spin (3/16, 19%), the FDA judged these to be questionable or negative.

## Discussion

This systematic review describes how spin has been explored in 35 reports, which were largely reviews of trials and observational studies with human subjects, across clinical areas. These reports documented various aspects related to the nature of spin in the included studies, which was also commonly referred to as ‘discordance between study results and conclusions’ or ‘overextrapolation’. In general, spin is prevalent in the biomedical literature, though this varies by study design, with the highest rates found in clinical trials. However, prevalence also appeared to vary according to the trial’s risk of bias and significance of primary outcomes. Spin manifests in diverse ways, which challenged investigators attempting to systematically identify and document instances of spin.

Spin was variably defined by investigators examining different bodies of biomedical research. As trials are designed to determine if an intervention is effective, authors may be motivated to interpret statistically nonsignificant findings in ways that still portray the intervention in a favourable light. In observational studies, study designs do not allow investigators to establish a causal relationship. Spin in these studies instead manifests as implying cause and effect to suggest a positive sequential relationship between an exposure and an outcome and to increase the perceived importance of the findings [40].

Spin is perhaps best understood in the context of RCTs with nonsignificant primary outcomes due to the development of a valid and reliable instrument by Boutron et al. [2], which has been applied across clinical areas. We identified 3 other valid instruments specifically for assessing spin in nonrandomised intervention studies [4], diagnostic accuracy studies [6], and systematic reviews [8]. However, researchers largely took an approach in which the nature of spin was prespecified and thus may not have fully explicated the full range of spin practices across study designs or clinical areas. This field could benefit from inductive approaches that aim to rigorously assess the diversity of spin practices, as well as evaluations of the effect of spin on those who rely upon biomedical evidence.

Our analysis identified several themes under which spin practices that occur across study designs and clinical areas can be grouped. These categories (inappropriate claims, inappropriate extrapolations or recommendations for clinical practice, selective reporting, and more robust or favourable data presentation) may be useful in educating researchers, peer reviewers, and editors about the various manifestations of spin, regardless of study type. These categories could also underpin instrument development focused on the assessment of spin that can be generalised beyond study design, which may be more useful to peer reviewers and editors of biomedical journals than tools specifically designed for clinical trials, for example.

Although investigators have hypothesised that a plethora of factors are related to the prevalence of spin, ranging from author characteristics to aspects of study design, there is very little evidence to suggest that any of these are related to the presence of spin. Industry sponsorship, which was the most common factor examined, was also not significantly associated with spin. Widening the investigation of factors contributing to spin from characteristics of individual authors or studies to the cultures and structures of research, which may incentivise or de-incentivise spin, would be instructive in developing strategies to mitigate the occurrence of spin in biomedical research.

To our knowledge, this is the first methodological systematic review investigating spin in published biomedical literature across a variety of fields. Thus, the aims were exploratory, and due to the heterogeneity of studies meeting the inclusion criteria, we were not able to fully answer questions related to the nature, prevalence, or implications of spin. Other methodological systematic reviews have been conducted with regards to publication bias [41], outcome reporting bias [39], funding bias [42], and selective reporting and inclusion of results [43]. Although the concept of spin draws on features of selective reporting of results, such as giving outcome data different prominence throughout different sections of a report [43], spin involves the additional aspect of interpretation bias. This systematic review highlights that further work is needed in the area of developing instruments and standards for assessing the occurrence of spin across different study designs. Little is known about the contextual factors that contribute to spin, and even less is known about the impact of spin on research, clinical practice, or policy environment.

Despite the lack of tools to assist with the identification of spin, there are a number of safeguards that can prevent spin. First, as routinely occurs, peer reviewers and journal editors check that abstract and manuscript conclusions are consistent with the study results, for inappropriate use of causal language, and for overgeneralisation. Second, clinical practice and public health guidelines should be developed based on systematic reviews to ensure that recommendations are founded on rigorous data and not misleading conclusions. Third, promoting fully open data or inviting published interpretation of published data from multiple researchers could mitigate the occurrence of spin. Finally, structural reforms within academia are needed to change research incentives and reward structures that emphasise ‘positive’ conclusions, including the pressure to publish and media attention.

Our review had a few key limitations. First, there are no predefined terms for spin, resulting in difficulty with formulating a comprehensive but specific search strategy. Our search strategy involved identifying possible words and phrases that could encompass the concept of spin in scientific research and exploring how potentially included papers were indexed in MEDLINE and Embase. Additionally, we hand-searched the reference lists of included reports to identify other potential articles. However, despite these measures, it is possible that reports were missed. Second, the included reports were heterogeneous; spin was investigated in numerous different ways across multiple study designs. As a result, it was only possible to descriptively analyse the characteristics of spin that were measured in most instances. Third, it is possible that some of the included reports that focused on the same area of research may have included the same studies. However, examination of the search strategies and included studies of the included reports (where provided) suggests that overlap is unlikely.

Despite these limitations, we conducted a comprehensive search for all studies investigating spin in the biomedical literature. We did not discover any reports that investigated spin in animal studies. As these studies often lay the groundwork for future interventions to be tested with human subjects, the presence of spin could contribute to the failure to translate scientific findings into clinical trials or human applications when results do not live up to their ‘hype’.

The reports included in our review noted some key limitations relevant to the investigation of spin, including the need to develop robust interpretive methodologies, as the assessment of spin is inherently open to interpretation and the thresholds for things like ‘significance’ are arbitrary and contextual. Future studies should consider more inductive and exploratory approaches, particularly when assessing spin in diverse study designs, as spin can manifest in variable ways. However, research that contributes to understanding how spin affects scientific, clinical, and policy decision-making, as well as the development of tools for scientists, peer reviewers, and editors, is needed.

### Conclusions

Spin in biomedical research is prevalent across a range of study designs, including trials, observational studies, diagnostic accuracy studies, and systematic reviews. Included reports examined and assessed spin in a variety of ways, and the definitions and spin practices identified may vary according to the study type investigated. Further research is required to develop more comprehensive and reproducible measures of spin across research fields. Further investigation of factors contributing to spin, particularly at the cultural and structural levels of research, is needed to develop ways of reducing spin. Editors and peer reviewers should be made aware of the widespread prevalence of spin and ways to avoid it in order to ensure accurate research interpretation and dissemination.

## Materials and methods

We conducted a methodological systematic review according to the PRISMA guidelines (S1 Text) [44].

### Inclusion and exclusion criteria

We broadly defined ‘spin’ as any reporting practices that distort the interpretation of results and mislead readers so that results are viewed in a more favourable light [2]. We searched for reports that included the measurement of spin in any of its forms as at least 1 stated outcome and provided quantitative data measuring spin. We included reviews, cross-sectional studies, cohort studies, and other empirical studies. We excluded editorials, perspectives, commentaries, and papers that examined spin in publications other than published biomedical literature, such as press releases or media reports. There were no limits placed on language or date of publication.

### Data sources and searching

The MEDLINE, PreMEDLINE, Embase, and Scopus (fields of Life Sciences and Health Sciences) databases were searched for articles published from 1946 (MEDLINE), 1974 (Embase), and 1960 (Scopus) through 24 November 2016. The search strategy for MEDLINE and Embase included combining (1) words and phrases that encompassed the concept of spin in biomedical research; and (2) the indexed term for research as a topic, which captured reports that investigated spin in published studies (S2 Text).

### Study screening and selection

One author (KC) performed the search and screened for relevant titles and abstracts for obvious exclusions (for example, ‘spin’ particles in physics articles). Both KC and QG independently assessed the 127 full texts for inclusion, with LB reviewing any discrepancies and disagreements. KC and QG independently searched the reference lists of included articles for additional papers during the process of duplicate data extraction.

### Data extraction

Two authors (KC and QG) independently extracted data into a collection form generated using REDCap electronic data capture tools hosted at The University of Sydney [45]. Data were collected on the following characteristics for each report: year of publication; journal name; funding source; author conflicts of interest; study design; and sample size. Data were also collected on the following characteristics regarding the included studies: field of research; time frame; definition of spin; location of spin; method of measuring spin; and all spin-related outcomes. We included all spin-related findings, whether or not they were explicitly presented as such, and extracted these findings verbatim. For example, not every report explicitly referred to spin (e.g., some reports measured ‘discordance between study results and conclusions’). Any discrepancies in data extraction were reviewed and discussed until consensus was reached.

### Assessing risk of bias

We categorised included reports by study design. Assessing risk of bias was not possible due to the heterogeneity in the study designs of our included reports and in the outcomes measured to assess spin. Furthermore, we did not wish to exclude any reports of low quality, due to the exploratory nature of this review.

### Synthesis of results

We calculated frequencies where possible for report and study characteristics. For unstructured data, we conducted a descriptive and thematic analysis with the goal of presenting the full range of findings.

We grouped the reports’ findings inductively according to spin-related outcome measures. This meant extracting all spin-related data reported in each of the included reports into an Excel spread sheet as ‘Findings.’ Then, we grouped these extracted data into categories based on shared characteristics; for example, all the frequency measures were grouped as ‘prevalence’ and any measure of the association between the occurrence of spin and an author, study, or reporting characteristic as ‘factors associated with spin’. The final categories included: how spin was defined, prevalence of spin, level of spin, practices used to spin results, and factors associated with spin. These categories were not predetermined but were expanded and added until all spin-related findings were accounted for.

We calculated the prevalence of spin by ascertaining whether each paper examined spin in the abstracts and/or main texts of original studies and recording or calculating the prevalence (x/*n*, percentage) of spin in the abstracts and main texts separately. The median prevalence and range were calculated for each study type.

When available and appropriate, quantitative data on the association of spin with study characteristics were combined by meta-analysis using ReviewManager 5.3 software (Cochrane Collaboration). Statistical heterogeneity was assessed using the *I*^2^ statistic, and a fixed-effect model was used.

## Supporting information

S1 Text PRISMA checklist. (DOCX)

S2 Text Systematic review search strategy. (DOCX)

## Abbreviations

FDA: United States Food and Drug Administration
RCT: randomised control trial
RR: risk ratio

## References

[1] Caulfield T, Ogbogu U. The commercialization of university-based research: Balancing risks and benefits. BMC Medical Ethics. 2015;16(1):1–7. doi: 10.1186/s12910-015-0064-2 26464028 10.1186/s12910-015-0064-2 PMC4605102

[2] Boutron I, Dutton S, Ravaud P, Altman DG. Reporting and interpretation of randomized controlled trials with statistically nonsignificant results for primary outcomes. JAMA. 2010;303(20):2058–64. doi: 10.1001/jama.2010.651 20501928 10.1001/jama.2010.651

[3] Horton R. The rhetoric of research. BMJ. 1995;310(6985):985–7. 7728037 10.1136/bmj.310.6985.985 PMC2549363

[4] Lazarus C, Haneef R, Ravaud P, Boutron I. Classification and prevalence of spin in abstracts of non-randomized studies evaluating an intervention. BMC Med Res Methodology. 2015;15:85 doi: 10.1186/s12874-015-0079-x 26462565 10.1186/s12874-015-0079-x PMC4604617

[5] Lockyer S, Hodgson R, Dumville JC, Cullum N. "Spin" in wound care research: the reporting and interpretation of randomized controlled trials with statistically non-significant primary outcome results or unspecified primary outcomes. Trials. 2013;14:371 doi: 10.1186/1745-6215-14-371 24195770 10.1186/1745-6215-14-371 PMC3832286

[6] Ochodo EA, de Haan MC, Reitsma JB, Hooft L, Bossuyt PM, Leeflang MM. Overinterpretation and misreporting of diagnostic accuracy studies: evidence of "spin". Radiology. 2013;267(2):581–8. doi: 10.1148/radiol.12120527 23360738 10.1148/radiol.12120527

[7] Prasad V, Jorgenson J, Ioannidis JP, Cifu A. Observational studies often make clinical practice recommendations: an empirical evaluation of authors' attitudes. J Clin Epidemiol. 2013;66(4):361–6.e4. doi: 10.1016/j.jclinepi.2012.11.005 23384591 10.1016/j.jclinepi.2012.11.005

[8] Yavchitz A, Ravaud P, Altman DG, Moher D, Hrobjartsson A, Lasserson T, et al A new classification of spin in systematic reviews and meta-analyses was developed and ranked according to the severity. J Clin Epidemiol. 2016. doi: 10.1016/j.jclinepi.2016.01.020 26845744 10.1016/j.jclinepi.2016.01.020

[9] Alasbali T, Smith M, Geffen N, Trope GE, Flanagan JG, Jin Y, et al Discrepancy between results and abstract conclusions in industry- vs nonindustry-funded studies comparing topical prostaglandins. Am J Ophthal. 2009;147(1):33–8.e2. doi: 10.1016/j.ajo.2008.07.005 18760766 10.1016/j.ajo.2008.07.005

[10] Altwairgi AK, Booth CM, Hopman WM, Baetz TD. Discordance between conclusions stated in the abstract and conclusions in the article: analysis of published randomized controlled trials of systemic therapy in lung cancer. J Clin Onc. 2012;30(28):3552–7. 10.1200/JCO.2012.41.8319 22649130

[11] Arunachalam L, Hunter IA, Killeen S. Reporting of randomized controlled trials with statistically nonsignificant primary outcomes published in high-impact surgical journals. Ann Surg. 2016 doi: 10.1097/SLA.0000000000001795 27257737 10.1097/SLA.0000000000001795

[12] Boutron I, Altman DG, Hopewell S, Vera-Badillo F, Tannock I, Ravaud P. Impact of spin in the abstracts of articles reporting results of randomized controlled trials in the field of cancer: the SPIIN randomized controlled trial. J Clin Onc. 2014;32(36):4120–6. 10.1200/JCO.2014.56.7503 25403215

[13] Brody BA, Ashton CM, Liu D, Xiong Y, Yao X, Wray NP. Are surgical trials with negative results being interpreted correctly? J Am Coll Surgeons. 2013;216(1):158–66. 10.1016/j.jamcollsurg.2012.09.015 PMC4165332 23177270

[14] Brown AW, Bohan Brown MM, Allison DB. Belief beyond the evidence: using the proposed effect of breakfast on obesity to show 2 practices that distort scientific evidence. Am J Clin Nutr. 2013;98(5):1298–308. doi: 10.3945/ajcn.113.064410 24004890 10.3945/ajcn.113.064410 PMC3798081

[15] Cofield SS, Corona RV, Allison DB. Use of causal language in observational studies of obesity and nutrition. Obesity Facts. 2010;3(6):353–6. doi: 10.1159/000322940 21196788 10.1159/000322940 PMC3280017

[16] Cordoba G, Schwartz L, Woloshin S, Bae H, Gotzsche PC. Definition, reporting, and interpretation of composite outcomes in clinical trials: Systematic review. BMJ. 2010;341(7769):381 http://dx.doi.org/10.1136/bmj.c3920. 10.1136/bmj.c3920 PMC2923692 20719825

[17] Djulbegovic B, Kumar A, Magazin A, Schroen AT, Soares H, Hozo I, et al Optimism bias leads to inconclusive results-an empirical study. J Clin Epidemiol. 2011;64(6):583–93. doi: 10.1016/j.jclinepi.2010.09.007 21163620 10.1016/j.jclinepi.2010.09.007 PMC3079810

[18] Fernandez Y Garcia E, Nguyen H, Duan N, Gabler NB, Kravitz RL. Assessing heterogeneity of treatment effects: Are authors misinterpreting their results? Health Services Res. 2010;45(1):283–301. 10.1111/j.1475-6773.2009.01064.x PMC2813449 19929962

[19] Gewandter JS, McKeown A, McDermott MP, Dworkin JD, Smith SM, Gross RA, et al Data interpretation in analgesic clinical trials with statistically nonsignificant primary analyses: an ACTTION systematic review. J Pain. 2015;16(1):3–10. doi: 10.1016/j.jpain.2014.10.003 25451621 10.1016/j.jpain.2014.10.003

[20] Hernandez AV, Pasupuleti V, Deshpande A, Thota P, Collins JA, Vidal JE. Deficient reporting and interpretation of non-inferiority randomized clinical trials in HIV patients: a systematic review. PLoS ONE. 2013;8(5):e63272 doi: 10.1371/journal.pone.0063272 23658818 10.1371/journal.pone.0063272 PMC3643946

[21] Jefferson T, Di Pietrantonj C, Debalini MG, Rivetti A, Demicheli V. Relation of study quality, concordance, take home message, funding, and impact in studies of influenza vaccines: systematic review. BMJ. 2009;338 doi: 10.1136/bmj.b354 19213766 10.1136/bmj.b354 PMC2643439

[22] Latronico N, Metelli M, Turin M, Piva S, Rasulo FA, Minelli C. Quality of reporting of randomized controlled trials published in Intensive Care Medicine from 2001 to 2010. Intensive Care Med. 2013;39(8):1386–95. doi: 10.1007/s00134-013-2947-3 23743522 10.1007/s00134-013-2947-3

[23] Le Fourn E, Giraudeau B, Chosidow O, Doutre MS, Lorette G. Study design and quality of reporting of randomized controlled trials of chronic idiopathic or autoimmune urticaria: review. PLoS ONE. 2013;8(8). doi: 10.1371/journal.pone.0070717 23940632 10.1371/journal.pone.0070717 PMC3733774

[24] Li LC, Moja L, Romero A, Sayre EC, Grimshaw JM. Nonrandomized quality improvement intervention trials might overstate the strength of causal inference of their findings. J Clin Epidemiol. 2009;62(9):959–66. Epub 2009/02/13. doi: 10.1016/j.jclinepi.2008.10.008 . 19211223 10.1016/j.jclinepi.2008.10.008

[25] Lieb K, Osten-Sacken Jvd, Stoffers-Winterling J, Reiss N, Barth J. Conflicts of interest and spin in reviews of psychological therapies: a systematic review. BMJ Open. 2016;6(4). doi: 10.1136/bmjopen-2015-010606 27118287 10.1136/bmjopen-2015-010606 PMC4853969

[26] Lumbreras B, Parker LA, Porta M, Pollan M, Ioannidis JP, Hernandez-Aguado I. Overinterpretation of clinical applicability in molecular diagnostic research. Clinical Chem. 2009;55(4):786–94. doi: 10.1373/clinchem.2008.121517 19233907 10.1373/clinchem.2008.121517

[27] Mathieu S, Giraudeau B, Soubrier M, Ravaud P. Misleading abstract conclusions in randomized controlled trials in rheumatology: Comparison of the abstract conclusions and the results section. Joint Bone Spine. 2012;79(3):262–7. doi: 10.1016/j.jbspin.2011.05.008 21733728 10.1016/j.jbspin.2011.05.008

[28] Patel SV, Chadi SA, Choi J, Colquhoun PHD. The use of "spin" in laparoscopic lower GI surgical trials with nonsignificant results: an assessment of reporting and interpretation of the primary outcomes. Diseases Colon and Rectum. 2013;56(12):1388–94. 10.1097/01.dcr.0000436466.50341.c5 24201393

[29] Patel SV, Van Koughnett JAM, Howe B, Wexner SD. Spin is common in studies assessing robotic colorectal surgery: An assessment of reporting and interpretation of study results. Diseases Colon and Rectum. 2015;58(9):878–84. 10.1097/DCR.0000000000000425 26252850

[30] Pocock SJ, Hughes MD, Lee RJ. Statistical problems in the reporting of clinical trials. A survey of three medical journals. NEJM. 1987;317(7):426–32. doi: 10.1056/NEJM198708133170706 3614286 10.1056/NEJM198708133170706

[31] Roest AM, de Jonge P, Williams CD, de Vries YA, Schoevers RA, Turner EH. Reporting bias in clinical trials investigating the efficacy of second-generation antidepressants in the treatment of anxiety disorders: a report of 2 meta-analyses. JAMA Pyschiatry. 2015;72(5):500–10. 10.1001/jamapsychiatry.2015.15 25806940

[32] Tricco AC, Tetzlaff J, Pham B, Brehaut J, Moher D. Non-Cochrane vs. Cochrane reviews were twice as likely to have positive conclusion statements: cross-sectional study. J Clin Epidemiol. 2009;62(4):380–6.e1. doi: 10.1016/j.jclinepi.2008.08.008 19128940 10.1016/j.jclinepi.2008.08.008

[33] Vedula SS, Goldman PS, Rona IJ, Greene TM, Dickersin K. Implementation of a publication strategy in the context of reporting biases. A case study based on new documents from Neurontin litigation. Trials. 2012;13:136 doi: 10.1186/1745-6215-13-136 22888801 10.1186/1745-6215-13-136 PMC3439687

[34] Vera-Badillo FE, Shapiro R, Ocana A, Amir E, Tannock IF. Bias in reporting of end points of efficacy and toxicity in randomized, clinical trials for women with breast cancer. Ann Oncology. 2013;24(5):1238–44. doi: 10.1093/annonc/mds636 23303339 10.1093/annonc/mds636

[35] Wilson JR. Rhetorical strategies used in the reporting of implantable defibrillator primary prevention trials. Am J Cardiology 2011;107(12):1806–11. 10.1016/j.amjcard.2011.02.320 21482417

[36] Yank V, Rennie D, Bero LA. Financial ties and concordance between results and conclusions in meta-analyses: retrospective cohort study. BMJ. 2007;335(7631):1202–5. doi: 10.1136/bmj.39376.447211.BE 18024482 10.1136/bmj.39376.447211.BE PMC2128658

[37] You B, Gan HK, Pond G, Chen EX. Consistency in the analysis and reporting of primary end points in oncology randomized controlled trials from registration to publication: a systematic review. J Clin Onc. 2012;30(2):210–6. 10.1200/JCO.2011.37.0890 22162583

[38] Ridker P, Torres J. Reported outcomes in major cardiovascular clinical trials funded by for-profit and not-for-profit organizations: 2000–2005. JAMA. 2006;295(19):2270–4. doi: 10.1001/jama.295.19.2270 16705108 10.1001/jama.295.19.2270

[39] Dwan K, Gamble C, Williamson PR, Kirkham JJ. Systematic review of the empirical evidence of study publication bias and outcome reporting bias—an updated review. PLoS ONE. 2013;8(7):e66844 doi: 10.1371/journal.pone.0066844 23861749 10.1371/journal.pone.0066844 PMC3702538

[40] Martin W. Making valid causal inferences from observational data. Preventive Vet Med. 2014;113(3):281–97. doi: 10.1016/j.prevetmed.2013.09.006 24113257 10.1016/j.prevetmed.2013.09.006

[41] Dubben HH, Beck-Bornholdt HP. Systematic review of publication bias in studies on publication bias. BMJ. 2005;331(7514):433–4. doi: 10.1136/bmj.38478.497164.F7 15937056 10.1136/bmj.38478.497164.F7 PMC1188109

[42] Lundh A, Sismondo S, Lexchin J, Busuioc OA, Bero L. Industry sponsorship and research outcome. Cochrane Database Syst Rev. 2012;12 doi: 10.1002/14651858.MR000033.pub2 23235689 10.1002/14651858.MR000033.pub2

[43] Page MJ, McKenzie JE, Kirkham J, Dwan K, Kramer S, Green S, et al Bias due to selective inclusion and reporting of outcomes and analyses in systematic reviews of randomised trials of healthcare interventions. Cochrane Database Syst Rev. 2014;(10):Mr000035 doi: 10.1002/14651858.MR000035.pub2 25271098 10.1002/14651858.MR000035.pub2 PMC8191366

[44] Moher D, Liberati A, Tetzlaff J, Altman DG, The PG. Preferred Reporting Items for Systematic Reviews and Meta-Analyses: The PRISMA Statement. PLoS Med. 2009;6(7):e1000097 doi: 10.1371/journal.pmed.1000097 19621072 10.1371/journal.pmed.1000097 PMC2707599

[45] Harris PA, Taylor R, Thielke R, Payne J, Gonzalez N, Conde JG. Research electronic data capture (REDCap)—A metadata-driven methodology and workflow process for providing translational research informatics support. J Biomed Informatics. 2009;42(2):377–81. http://dx.doi.org/10.1016/j.jbi.2008.08.010. 10.1016/j.jbi.2008.08.010 PMC2700030 18929686

