# Supplementary material for: ‘Spin’ in published biomedical literature: A methodological systematic review
Source: PLoS Biol. 2017 Sep 11;15(9):e2002173. doi: 10.1371/journal.pbio.2002173 (PMC5593172; doi:10.1371/journal.pbio.2002173)
Supplement: S2 Text — (DOCX) [file pbio.2002173.s002.docx]

**Appendix S1. Search strategy of electronic databases**

Search of MEDLINE conducted on 24 November 2016

1. (outcome$ and (report$ adj1 bias$)).mp.

2. spin.mp.

3. exp *"Bias (Epidemiology)"/

4. discordan*.mp.

5. (concordan$ not (concordance adj ind$)).mp.

6. ((nonsignificant$ or non-significan$ or ("not" adj significant)) adj2 result$).mp.

7. (report$ adj30 interpret$).mp.

8. exp *epidemiologic study characteristics as topic/

9. 1 or 2 or 3 or 4 or 5 or 6 or 7

10. 8 and 9

Search of PreMEDLINE conducted on 24 November 2016

1. (outcome$ and (report$ adj1 bias$)).mp.

2. spin.mp.

3. discordan*.mp.

4. (concordan$ not (concordance adj ind$)).mp.

5. ((nonsignificant$ or non-significan$ or ("not" adj significant)) adj2 result$).mp.

6. (report$ adj30 interpret$).mp.

7. 1 or 2 or 3 or 4 or 5

8. 6 and 7

Search of Embase conducted on 24 November 2016

1. (outcome$ and (report$ adj1 bias$)).mp.

2. spin.mp.

3. exp *statistical bias/

4. discordan*.mp.

5. (concordan$ not (concordance adj ind$)).mp.

6. ((nonsignificant$ or non-significan$ or ("not" adj significant)) adj2 result$).mp.

7. (report$ adj30 interpret$).mp.

8. exp *research/

9. 1 or 2 or 3 or 4 or 5 or 6 or 7

10. 8 and 9

Search of Scopus conducted on 24 November 2016

( TITLE-ABS-KEY ( ( report*  W/30  interpret* ) )  AND  SUBJAREA ( mult  OR  agri  OR  bioc  OR  immu OR  neur  OR  phar  OR  mult  OR  medi  OR  nurs  OR  vete  OR  dent  OR  heal ) )  AND  ( ( TITLE-ABS-KEY ( ( outcome*  AND  ( report*  W/1  bias* ) ) )  AND  SUBJAREA ( mult  OR  agri  OR  bioc  OR  immu OR  neur  OR  phar  OR  mult  OR  medi  OR  nurs  OR  vete  OR  dent  OR  heal ) )  OR  ( TITLE-ABS-KEY ( ( spin ) )  AND  SUBJAREA ( mult  OR  agri  OR  bioc  OR  immu  OR  neur  OR  phar  OR  mult  OR medi  OR  nurs  OR  vete  OR  dent  OR  heal ) )  OR  ( TITLE-ABS-KEY ( ( discordan* ) )  AND SUBJAREA ( mult  OR  agri  OR  bioc  OR  immu  OR  neur  OR  phar  OR  mult  OR  medi  OR  nurs  OR vete  OR  dent  OR  heal ) )  OR  ( ( TITLE-ABS-KEY ( ( concordan* ) )  AND  SUBJAREA ( mult  OR  agri OR  bioc  OR  immu  OR  neur  OR  phar  OR  mult  OR  medi  OR  nurs  OR  vete  OR  dent  OR  heal ) ) AND NOT  ( TITLE-ABS-KEY ( ( concordance  PRE/0  ( "INDEX"  OR  indices ) ) )  AND  SUBJAREA ( mult OR  agri  OR  bioc  OR  immu  OR  neur  OR  phar  OR  mult  OR  medi  OR  nurs  OR  vete  OR  dent OR  heal ) ) )  OR  ( TITLE-ABS-KEY ( ( ( nonsignifican*  OR  non-significan*  OR  ( not  PRE/0 significant ) )  PRE/2  result* ) )  AND  SUBJAREA ( mult  OR  agri  OR  bioc  OR  immu  OR  neur  OR phar  OR  mult  OR  medi  OR  nurs  OR  vete  OR  dent  OR  heal ) ) )
